# Nutrient Composition and Fatty Acid and Protein Profiles of Selected Fish By-Products

**DOI:** 10.3390/foods9020190

**Published:** 2020-02-14

**Authors:** Aikaterini Kandyliari, Athanasios Mallouchos, Nikos Papandroulakis, Jaya Prakash Golla, TuKiet T. Lam, Aikaterini Sakellari, Sotirios Karavoltsos, Vasilis Vasiliou, Maria Kapsokefalou

**Affiliations:** 1 Unit of Human Nutrition, Department of Food Science and Human Nutrition, Agricultural University of Athens, 11855 Athens, Greece; kkandyliari@aua.gr (A.K.); amallouchos@aua.gr (A.M.); 2 Institute of Marine Biology, Biotechnology and Aquaculture, Hellenic Center for Marine Research, 71003 Heraklion, Greece; npap@hcmr.gr; 3 Department of Environmental Health Sciences, Yale School of Public Health, New Haven, CT 06510, USA; jayaprakashgolla@gmail.com (J.P.G.); vasilis.vasiliou@yale.edu (V.V.); 4 Department of Molecular Biophysics and Biochemistry, Yale University, New Haven, CT 06520, USA; tukiet.lam@yale.edu; 5 Keck MS & Proteomics Resource, WM Keck Biotechnology Resource Laboratory, New Haven, CT 06510, USA; 6 Laboratory of Environmental Chemistry, Department of Chemistry, National and Kapodistrian University of Athens, Panepistimioupolis, Zografou, 15784 Athens, Greece; esakel@chem.uoa.gr (A.S.); skarav@chem.uoa.gr (S.K.)

**Keywords:** fish waste, circular economy, sea bream, meagre, nutrient content, proximate composition, proteomics, aquaculture

## Abstract

Processing of fish in aquaculture generates considerable amounts of by-products that remain underused and/or unexploited. We evaluated the nutritive content of fish by-products (head, gills, intestines, trimmings, bones, and skin) from meagre and gilthead sea bream fish species reared in Greece in order to estimate their nutritional value for future development of high added-value products. The proximate composition of the fish samples (total protein, total lipid, ash, moisture, and macro-element content) was determined using the Association of Official Analytical Chemists (AOAC) and International Organization for Standardization (ISO) official methods. The content of fatty acids was determined using capillary gas chromatography, and the protein profile was estimated employing scientific orbitrap mass spectrophotometer methodology. The nutrient composition of fish by-products presented fluctuations among the different by-products. Skin was the most significant protein source, trimmings and bones were high in calcium, and the head, intestines, and bones were a good source of lipids. The most abundant lipid acids found in by-products were oleic, palmitic, linoleic, and eicosenoic acids, whereas the most abundant proteins were adenosine triphosphate (ATP) synthase subunit epsilon, mitochondrial nicotinamide adenine dinucleotide (NADH) dehydrogenase, and mitochondrial cytochrome b-c1 complex subunit 8. These data suggest that by-products constitute valuable sources of nutrients and could therefore be exploited in accordance with the principles of a circular economy.

## 1. Introduction

Processing of fish generates by-products (bones, skin, head, viscera), which may constitute up to 15% of fish weight when gutting and scaling or even up to 70% of fish weight when filleting [1]. According to Food and Agriculture Organization of the United Nations (FAO) report, 9.1 million tons of fish waste are estimated to be discarded annually [2]. Thus, fish by-products have become a global concern and also threaten the sustainability of fish aquaculture. Fish by-products are, in most cases, either incorporated into animal feed or biofuels, i.e., low added-value products, or incinerated and discarded, thus increasing the energy consumption, financial cost, and environmental impact of their management process [3]. 

There are emerging exploitation opportunities for fish by-products. Producers’ demand for natural ingredients of high nutritional value is currently being reported in accordance with the public’s nutritional requirements [4]. These natural ingredients may derive from food by-products produced along the agri-food chain. A database containing the nutritional composition and alternative uses of food waste [5] demonstrates that in order to valorize underutilized food materials, the complete characterization of their nutrient composition and properties is required. Fish by-products may be an important source for such ingredients. For example, protein, hydrolysates, peptides, and fatty acids [6,7,8,9,10,11] have been determined in fish by-products and linked to antihypertensive, antioxidant, and antimicrobial activities [12,13,14,15].

Research on the exploitation of fish by-products for the development of high added-value products, such as novel foods, constitutes a financial and nutritional challenge which will significantly ameliorate the aquaculture sector’s performance, in regard to sustainability. To address this challenge, a systematic approach to the study of fish-products is required with country-specific information in order to address a variety of factors, such as fish species, size, and environmental conditions that affect nutrient composition [16,17]. 

There are limited data from aquaculture units in Greece, despite the size and penetration of the industry. Greek aquaculture exports 80% of its production to 32 countries [18]. Species of interest include gilthead sea bream (*Sparus aurata*), sea bass (*Dicentrarchus labrax*), and meagre (*Argyrosomus regius*) [19]. Limited data on the fatty acid content of farmed meagre fillet and its by-products [20] and gilthead sea bream and sea bass fillet [21] are available. Thus, there is a gap in the nutritional content of the by-products of those fish species, which creates a barrier to their potential exploitation.

The purpose of this study was to evaluate the nutritional content of fish by-products from meagre and gilthead sea bream produced during fish filleting procedures to provide insight into their possible future uses. Specifically, in a series of filleting by-products from meagre and gilthead sea bream fishes of different size classes, the contents of total protein, fat, hydration, ash, and main minerals were determined, and their fatty acid and protein profiles were characterized.

## 2. Materials and Methods

### 2.1. Chemicals and Reagents

All chemicals and reagents were of analytical grade and were purchased from Sigma–Aldrich (Merck KGaA, Darmstadt, Germany) and Merck (Merck KGaA, Darmstadt, Germany). 

### 2.2. Fish Sample Collection and Preparation

A total of 36 individuals of meagre and 60 individuals of gilthead sea bream, of two different size classes (small and large), were obtained from the pilot-scale cage farm of the Hellenic Centre for Marine Research (HCMR) in Crete, Greece, in June 2017. The size class (small and large) was defined by the weight of the fish; small fishes were assumed to have a body weight less than 300 g for meagre and less than 200 g for gilthead sea bream. Fishes were fed with commercial feed pellets (Irida S.A., Arta, Greece) consisting of fishmeal, maize gluten, fish oil, sunflower flour, and soy flour. The chemical composition of the pellets was as follows: 43.0% crude protein, 17.0% crude fat, 2.7% crude fiber, 8.2% ash, 8.5% moisture, 1.15% total P, 1.5% total Ca, Vit. A 10.000 IU, Vit. D3 2.000 IU, Vit. E 400 mg, Vit. C 500 mg, choline 750 mg. Among the 36 individuals of meagre, 6 large fish had a mean body weight of 1256.45 ± 232.32 g and 30 small fish had a mean body weight of 235.76 ± 38.45 g. Among 60 individuals of gilthead sea bream, 16 large fish had a mean body weight of 403.47 ± 72.92 g and 44 small fish had a mean body weight 160.16 ± 30.79 g. After measuring the morphometric characteristics of each fish, we examined six different by-products: head, gills, intestines, trimmings, bones, and skin. These different by-products were kept separately in tightly packaged polypropylene bags at −80 °C. To perform the analysis, the same types of by-product categories from different individuals were pooled together to obtain a sample of approximately 100 g, following the methodology of previous studies [22,23,24,25,26]. For this purpose, more than 72 pooled samples of fish by-products were obtained (2 fish species, 2 sizes of each species, 6 fish by-products, 3–4 individual pool replicates). Specifically, pooling of by-products was performed according to their six categories, species and size class, providing triplicates for a total of 24 different sample categories. All samples were lyophilized for 48 h (Telstar Cryodos 50, Telstar, Terrassa, Spain) and then homogenized with a knife mill for 10 s at a revolution speed of 10000 rpm (Retsch ZM 200, Retsch, Hahn, Germany). Homogenized powders were stored at −80 °C until further analysis.

All fishes were reared at the pilot scale in the growing facilities of HCMR. The installations are licensed facilities for operations of breeding and experimentation use of fish issued by the Region of Crete, General Directorate of Agricultural and Veterinary No 3989/01.03.2017. The breeding and experimental facilities were registered with the following approval codes: EL91-BIObr-03 and EL91-BIOexp-04. The fish samples were collected from groups reared under normal conditions and after harvesting, were euthanized using ice water. The personnel that performed the sampling has degrees accredited by the Federation of European Laboratory Animal Science Associations (FELASA) on the “care and use of laboratory animals” for persons carrying out procedures on animals, designing procedures and projects, taking care of animals, and killing animals.

### 2.3. Proximate Composition

The proximate composition of the different fish by-product samples was determined using the following Association of Official Analytical Chemists (AOAC) [27] and International Organization for Standardization (ISO) methods. The moisture content of samples was determined using a vacuum oven (AOAC 952.08) prior to sample lyophilization. The samples were kept in properly sealed containers and carefully resuspended after thawing to avoid any losses. Total ash determination was conducted according to AOAC 938.08, and the total fat content according to AOAC 948.15. The nitrogen content was determined using an automated Kjeldahl apparatus (Kjeltec 8100, Foss Analytical, Hilleroed, Denmark) following the procedure described in ISO 5983-2:2005 [28]. The total protein content was calculated by multiplying the nitrogen content by a conversion factor of 6.25. Total carbohydrates were determined by subtracting the sum of the ash, fat, and protein contents from 100 [29]. All analyses were conducted in triplicates. The results for the moisture content are expressed as g/100 g of sample, whereas those for ash, protein, fats, and carbohydrates as g/100 g of dry matter.

### 2.4. Mineral Analysis

Macro-elements (Ca, Na, K, Mg) were determined in the different fish by-product samples. The lyophilized samples were digested with HNO_3_ 65% supra pure, and H_2_O_2_ 30% was subsequently added according to the procedures described in Milanov et al. [30]. Measurement of the macro-elements K, Na, Ca, and Mg was performed by atomic emission spectrometry, employing a Varian SpectrAA 200 (Varian, Mulgrave, Australia) instrument. All analyses were conducted in triplicate. The results are expressed as mg/g of dry matter. The percentages of recommended dietary allowances (RDA) and adequate intake (AI) were calculated based on the suggestions of the European Parliament and of the Council [31] and in accordance with the study of Montowska et al. [32].

### 2.5. Preparation of Samples for Fatty Acid and Protein Profile Analyses

For the determination of protein and fatty acid profiles, lyophilized samples of heads, skin, bones, trimmings, and gills were pooled together according to the two different species. The intestines were excluded from the pooled samples in order to minimize the variation in the case of non-fully fastened fishes. The nutrient profiles of the two final pooled samples of meagre and gilthead sea bream by-products were further analyzed and characterized according to the following methods.

### 2.6. Fatty Acid Profile

Fatty acids were determined in the above samples according to AOAC 996.06. Fat was extracted from fish samples using acidic hydrolysis, and after methylation, the fatty acid methyl esters (FAMEs) were quantitatively measured by capillary gas chromatography (Simadzu GC-2010 Plus, Simadzu Corporation, Kyoto, Japan) against a triglyceride (triundecanoin C11:0) internal standard. Separation of FAMEs was accomplished on a DB-wax capillary column (30 m × 0.25 mm i.d., 0.25 μm film thickness, Agilent). Helium was used as a carrier gas at a constant linear velocity of 30 cm/s. The split ratio was set at 1/10. Oven temperature was maintained at 100 °C for 1 min, programmed at 25 °C/min to 100 °C and after 1 min, it was raised to 240 °C at a rate of 5 °C/min and held for 2 min. Then, the temperature was raised to 250 °C at a rate of 5 °C/min and held for 10 min. Inlet and Flame Ionization Detector (FID) temperatures were set to 250 °C and 270 °C, respectively. Peak identification and response factor calculation were accomplished using a FAME standard mixture (Supelco 37 Component FAME mix, Sigma–Aldrich, Darmstadt, Germany). Calculations were performed according to AOAC 996.06. The results were expressed as g/100 g of dry matter. 

### 2.7. Protein Profile

For the determination of their protein profile, the above pooled by-product samples were dissolved in radioimmunoprecipitation assay (RIPA) buffer, supplemented with complete protease and phosphatase inhibitor cocktail. Then, the specimens (*n* = 3 for each pooled sample) were sonicated at 15 s bursts three times with a digital sonifier (Branson Ultrasonics, Branson, Danbury, Connecticut). The homogenate was then centrifuged at 16,000× *g* for 10 min at 4 °C, and 150 µL of the supernatant was added to methanol:chloroform (at ratio of 4:3 %*v*/*v*) for protein precipitation. Following vortex and centrifugation (16,000× *g* for 1 min), the supernatant was carefully discarded and an additional 400 µL of methanol was added. The mixture was centrifuged at 16,000× *g* for 2 min, and methanol was carefully discarded. The pellet was dissolved in 82 μL of 8 M urea in 0.4 M NH_4_HCO_3_ solution and then measured with a NanoDrop (2000/2000c Thermo Scientific, Fisher Scientific, Wilmington, DC, USA) to obtain the same amount of total protein from all the samples. Dithiothreitol (DTT) was then added to the resultant sample at a ratio of 10:1 %*v*/*v* (sample:DTT), and the mixture was incubated in the dark in an oven (Isotemp Incubator, Fischer Scientific, Wilmington, DE, USA) at 37 °C for 30 min. Subsequently, 8 μL of iodoacetamide (100 mM) was added to alkylate the sample, which was further diluted with mass spectrometry grade H_2_O in order to adjust the urea concentration to lower than 2 M. The sample was then enzymatically digested with Lys C (at 1:50 enzyme:protein ratio) at 37 °C for 16 h. The digestion continued with trypsin (at a 1:50 enzyme:protein ratio), and the sample was left in the incubator at 37 °C for 7 h. The digestion reaction was quenched with trifluoroacetic acid at a final 10% concentration, and the sample was then desalted with C18 UltraMicroSpin columns (The Nest Group Inc., Southborough, MA, USA) according to the manufacturer’s protocol. The eluate was subsequently dissolved in a mixture of 98% mass spectrometry-grade H_2_O, 0.1% formic acid, and 2% acetonitrile. Peptide concentration was determined for all samples and diluted accordingly to 0.05 μg/μL with 0.1% FA; then 5 μL of each sample was loaded onto the column for Liquid Chromatography with Mass Spectrometry (LC MS/MS ) analyses. Data collection for label-free quantitation (LFQ) proteomics was carried out on a mass spectrometer (Thermo Scientific Orbitrap Fusion) connected to a Ultra-performance Liquid Chromatography (UPLC) system (Waters nano ACQUITY) equipped with a Waters Symmetry^®^ C18 180 μm × 20 mm trap column and a 1.7-μm, 75 μm × 250 mm nano ACQUITY UPLC column (35 °C). Data processing of collected LFQ was performed similarly to the methodology published by the Keck Mass Spectrometry & Proteomics Resource [33]. For the addition of quantitative information to our proteomic results, the Exponentially Modified Protein Abundance Index (emPai) score was employed, which is proportional to the protein content of a protein sample [34].

### 2.8. Statistical Analysis

Statistical analysis was performed using the SPSS package, version 16.1 (SPSS Inc, Chicago, IL, USA). We deemed statistical significance at α = 0.05. Morphometric characteristics of the different fish as well as the percentage of weight of the different by-products are expressed as the mean ± standard deviation (SD); for these variables, normality was confirmed graphically with histograms (Figures S1–S6, Supplementary Material), and the differences between two different size classes (large and small) and two different species (meager and gilthead sea bream) were tested using Student’s *t*-test (unpaired analysis). The nutrient content is presented as the mean ± SD, and the non-parametric Mann–Whitney test was used to statistically compare values between two groups due to the small sample size. 

## 3. Results

The studied sample consisted of 36 and 60 individuals of meagre and gilthead sea bream, respectively. They were classified according to their body size into large and small fishes. Specifically, the meagre sample consisted of 6 large individuals of mean length 53.8 ± 3.9 cm, and 30 small individuals of mean length 26.9 ± 1.8 cm. Accordingly, the gilthead sea bream sample consisted of 16 large fish of mean length 28.3 ± 1.7 cm and 44 small fish of mean length 21.4 ± 1.5 cm.

Table 1 summarizes the six different by-products (head, gills, intestines, trimmings, bones, skin) as a percentage of the total weight for the two different species and the two different size classes. Comparing the sizes of the fish, no differences were observed for the percentage of head, gills, bones, and skin. However, the percentage of intestines relative to the total fish weight was lower in large fishes (4.78 ± 0.84%) compared to small ones (6.44 ± 1.33%), (*p* < 0.001). The percentage of trimmings relative to the total fish weight was 1.79 ± 0.69% in small fishes, exceeding the corresponding value for large fishes (1.64 ± 0.18%), (*p* = 0.007). No statistically important differences were observed between the two different size classes for the total by-product percentage. When comparing the two species, no differences were observed for the percentage of head, gills, intestines, and skin. However, the percentage of trimmings was lower in the meagre (1.48 ± 0.30%) compared to the gilthead sea bream (1.94 ± 0.61%), (*p* < 0.001), whereas that of bones was higher in meagre (7.65 ± 1.60%) than in gilthead sea bream (5.00 ± 0.66%), (*p* < 0.001). Finally, the total by-product percentage was different between the two fish species (*p* < 0.001); meagre by-product yield percentage was higher (43.06 ± 3.24%) than that of gilthead sea bream (38.26 ± 1.90%).

Table 2 present the nutrient composition (mean values) of the by-product samples (head, gills, intestines, trimmings, bones, skin) of the two size classes (large and small) for meagre and gilthead sea bream. No statistically significant differences, either between fish species or size classes, were observed for the different by-products based on the measured moisture, ash, total fat, total protein, carbohydrate, calcium, sodium, potassium, and magnesium contents. The mean values on a dry weight basis of all fish by-products for protein, fat, and ash contents were calculated. Specifically, the mean protein content was 44.27 ± 13.35%, the mean fat content was 26.48 ± 15.97%, and the mean ash content was 20.78 ± 14.44%.

The content of minerals, i.e., calcium (Ca), potassium (K), magnesium (Mg), and sodium (Na), in the different by-products is presented in Table 3. No statistical differences were observed between the two different size classes or between the two species with respect to the different minerals. Percentages of RDA and AI for Ca, K, Mg, and Na were calculated for all the different by-products for the content of 100 g of dried sample. By-products such as trimmings presented a valuable source of Ca, with RDA percentage reaching 466% for meager and 424% for gilthead sea bream fishes. The RDA percentage of Na did not exceed 39% for meagre and 45% for gilthead sea bream. Skin was a source of potassium, i.e., reaching 20% and 18% for meagre and gilthead sea bream, respectively. The Mg percentage was 110% of the RDA for meagre gill samples and 104% for gilthead sea bream trimming samples. 

The fatty acid content of the pooled samples from by-products of meagre and gilthead sea bream fishes is shown in Table 4. The majority of the fatty acid concentrations were statistically different between the two fish species. The most abundant fatty acids in the by-products from both fish species were oleic (18:1), palmitic (16:0), linoleic acid (18:2), and eicosenoic (20:1) acids. The total fatty acid content as well as the content of saturated, monounsaturated, and polyunsaturated fatty acids was significantly higher in the gilthead sea bream by-products.

Mean data of the proteomic profile of meagre and gilthead sea bream fish by-product samples are presented in Table 5. The determination of the abundance of each protein was derived through the application of the emPAI score [34]. The most abundant proteins identified in meagre by-products were adenosine triphosphate (ATP) synthase subunit epsilon, mitochondrial nicotinamide adenine dinucleotide (NADH) dehydrogenase 1 beta subcomplex subunit 1, 60S ribosomal protein L35a, cytochrome c oxidase, and mitochondrial cytochrome c. For gilthead sea bream by-products, the most abundant proteins were mitochondrial cytochrome b-c1 complex subunit 8, mitochondrial cytochrome c oxidase subunit 6B1, and NADH dehydrogenase, with an emPAI score higher than 0.85.

## 4. Discussion

The exploitation of fish by-products may support the sustainability of aquaculture. High by-product yields signify that a large part of fish is discarded and remains unexploited. In our study, this yield was estimated to be 43% for meagre and 38% for gilthead sea bream. Some studies have reported greater amounts of discards, i.e., up to 70–85% [15,35], while for finfish species such as sea bream, cod, tuna, etc., the discards can reach 40–65% [36,37,38,39]. The discards mainly include muscle-trimmings (15–20%), skin and fins (1–3%), bones (915%), heads (9–12%), viscera (12–18%), and scales (5%) [40]. In the present study, these percentages were calculated as 1–2% for trimmings, 5–9% for bones, 6–7% for skin, 2–3% for scales, 5–7% for intestines, and 17–19% for heads. Small variations can be attributed to the different fish species as well as processing, i.e., manual or mechanical.

In the present study, data on the composition of fish by-products were obtained. The nutrient content of total protein, fat, ash, and minerals was demonstrated in fish by-products of all species, size classes, and fish part categories. The mean values calculated on a dry weight basis of all fish by-products for protein, fat, and ash contents are in accordance with previous studies on fish waste, estimating a total protein content between 49.22 and 57.92% and a total ash content between 21.79 and 30.16%; however, total fat was between 7.16 and 19.10%, which is slightly higher than our study [5,41,42]. The nutrient content of specific by-product categories is comparable to that of fish fillets. For meagre fillets, the protein content was 75% of dry matter, the fat content was 19%, and the ash content was 5%. Corresponding values for gilthead sea bream fillet were 57% protein, 40% fat, and 4% ash, calculated on dry matter [21]. Some variation between the different categories exists although the differences were not statistically significant in the present study. The study by Sinanoglou et al. [20] on the by-products of meagre fishes showed that the skin had the highest protein concentration, followed by the head.

In the study reported herein, another important finding is the detailed characterization of the fatty acid profile of fish by-products. For both species, oleic acid (18:1n-9) was the main monosaturated fatty acid (MUFA) and predominated all fatty acids, followed by icosenoic acid (20:1n-9) and eurucic acid (22:1). Palmitic acid (16:0) was the main saturated fatty acid (SFA). The principal polyunsaturated fatty acid (PUFA) was linoleic acid (18:2n-6). These five fatty acids accounted for approximately 75% and 71% of total fatty acids for meagre and gilthead sea bream, respectively. These findings are similar to those of studies conducted in Greece on gilthead sea bream by-product and fillet samples [20,25] and on meagre [43]. According to Costa et al., meagre fillet was also high in docosahexaenoic acid (22:6n-3 or DHA). However, this was not abundant in our fish by-product samples [44]. This can be due to possible differences in the fish diets.

To define the protein composition and content of meagre and gilthead sea bream by-products, we exploited MS analysis for complex protein identification [45]. Among the identified proteins, only ATP synthase subunit epsilon and cytochrome c oxidase, which were abundant in the meagre samples, were significantly different between the species. This may suggest that these proteins are characteristic of meagre by-products. In addition, the sequence of these proteins was compared with bioactive peptides already published [46]. Two of the peptides, 60S ribosomal protein L35a and mitochondrial cytochrome b-c1 complex subunit 8, that were characterized as abundant in meagre and gilthead sea bream by-products, were proved to have the sequence of leucine-tryptophan and valine-tyrosine, respectively. According to Sato et al., these peptides from wakame showed an antihypertensive effect [47].

The interpretation of our findings must consider the following limitations. The sampling was conducted in a specific month; therefore, seasonality is a factor not considered in our experimental design. Quantities of fish by-product samples were not adequate for the analyses of all the parameters examined in the cases of gills and trimmings and in many small-sized fish regarding heads, intestines, bones, and skins. Pooling of samples masks the potential variations between individuals [48]. Studying the different by-products individually would demonstrate variations among different aquaculture units, seawater environments, and processing procedures.

## 5. Conclusions

Our results suggest that fish by-products constitute an important and nutritionally valuable source of proteins, fatty acids, and minerals since their composition is similar to those of fish fillet and other food products recommended for consumption. Each by-product category has specific nutritional features; thus, its potential exploitation depends on the desired specific nutritional characteristics of the products that we wish to formulate. In the present study, skin had the highest protein content, trimmings and bones were rich in calcium, and the head, intestines, and bones were a good source of lipids. However, these results may vary between different fish species and conditions of cultivation. Systematic information on different fish species and by-products is therefore necessary for the detailed and accurate evaluation of their exploitation.

## Figures and Tables

**Table 1:** Percentage weight ^1^ of the six different by-products (intestines, gills, trimmings, head, skin) of meagre and gilthead sea bream in two different size classes.

g By-Product/100 g Fish	Large Meagre (*n* = 6)		Small Meagre (*n* = 30)		Large Gilthead Sea Bream (*n* = 16)		Small Gilthead Sea Bream (*n* = 44)		P_1_	P_2_
	Mean	SD	Mean	SD	Mean	SD	Mean	SD		
Head	18.74	3.27	17.09	1.74	18.49	1.42	16.70	1.27	0.09	0.61
Gills	3.18	0.31	3.01	0.32	2.54	0.27	2.42	0.22	0.58	0.38
Intestines	5.07	0.53	7.44	0.95	4.49	0.90	5.44	0.87	<0.001	0.08
Trimmings	1.65	0.19	1.32	0.29	1.62	0.19	2.27	0.63	0.007	<0.001
Bones	8.76	3.09	6.53	0.76	4.95	0.47	5.04	0.72	0.50	<0.001
Skin	6.25	1.68	7.06	1.06	6.35	1.04	6.22	0.99	0.38	0.43
Sum	43.65	4.84	42.46	2.41	38.45	2.06	38.07	2.08	0.98	<0.001

^1^ Results are presented as the mean (SD) for normally distributed variables; P_1_ and P_2_ denote statistical significance between the two different size classes and between the two fish species, respectively; *p*-values derived through independent Student’s *t*-test with α = 0.05.

**Table 2:** Mean data for the nutrient composition of by-product samples (head, gills, intestines, trimmings, bones, skin) of meagre and gilthead sea bream fishes in two different size classes (large and small).

By-Product Nutrient Composition																									
**Meagre**	(g/100 g) *	**Head**				**Gills**				**Intestines**				**Trimmings**				**Bones**				**Skin**			
		**Large**		**Small**		**Large**		**Small**		**Large**		**Small**		**Large**		**Small**		**Large**		**Small**		**Large**		**Small**	
		**Mean**	**SD**	**Mean**	**SD**	**Mean**	**SD**	**Mean**	**SD**	**Mean**	**SD**	**Mean**	**SD**	**Mean**	**SD**	**Mean**	**SD**	**Mean**	**SD**	**Mean**	**SD**	**Mean**	**SD**	**Mean**	**SD**
	Moisture	64.0	0.5	68.9	0.7	68.3	0.2	74.3	0.4	73.0	0.2	59.2	0.4	63.1	0.7	57.0	0.1	63.2	0.7	40.6	0.2	58.4	0.3	65.3	0.2
	Ash	20.95	0.37	21.27	1.04	15.59	0.98	19.18	0.32	4.77	0.08	2.25	0.05	49.12	1.91	48.51	2.55	21.00	0.58	23.30	1.23	20.24	0.53	15.23	1.06
	Protein	40.41	0.16	47.50	1.25	45.62	0.16	48.46	0.32	59.62	0.08	29.79	0.64	45.87	0.91	45.98	1.92	32.07	0.25	36.41	0.13	75.16	1.87	75.15	0.03
	Fat	28.88	1.60	23.34	0.74	19.71	1.33	21.31	0.45	17.09	0.19	54.05	4.94	3.00	1.50	4.35	0.69	34.96	0.10	31.07	2.10	6.12	0.42	9.61	1.60
	Carbohydrates	9.76	1.65	7.89	1.78	19.08	1.66	11.05	0.63	18.52	0.22	13.91	4.98	2.01	2.59	1.16	3.27	11.97	0.64	9.22	2.44	1.01	0.01	0.99	0.01
**Gilthead Sea Bream**	(g/100 g) *	**Head**				**Gills**				**Intestines**				**Trimmings**				**Bones**				**Head**			
		**Large**		**Small**		**Large**		**Small**		**Large**		**Small**		**Large**		**Small**		**Large**		**Small**		**Large**		**Small**	
		**Mean**	**SD**	**Mean**	**SD**	**Mean**	**SD**	**Mean**	**SD**	**Mean**	**SD**	**Mean**	**SD**	**Mean**	**SD**	**Mean**	**SD**	**Mean**	**SD**	**Mean**	**SD**	**Mean**	**SD**	**Mean**	**SD**
	Moisture	57.3	0.7	62.4	0.2	66.6	0.3	62.9	0.8	67.1	1.0	57.15	0.5	48.6	0.1	53.1	0.2	53.3	0.7	74.5	0.8	53.0	0.5	61.2	0.1
	Ash	18.11	1.24	21.39	1.33	16.60	0.40	17.49	0.30	3.57	0.06	2.62	0.07	45.76	2.29	47.26	0.73	26.62	0.10	27.70	0.58	6.02	0.17	4.36	0.17
	Protein	32.40	0.45	37.19	0.67	31.49	0.42	38.50	1.47	37.23	0.75	26.87	0.35	41.85	1.00	45.10	2.30	34.02	0.98	40.74	1.57	43.16	0.89	49.67	0.11
	Fat	37.08	4.19	28.76	0.47	37.46	1.16	26.69	0.23	43.19	0.35	55.12	0.98	5.45	0.09	4.09	0.33	30.56	0.11	21.47	0.54	46.39	3.45	45.94	0.54
	Carbohydrates	12.41	4.39	12.66	1.56	14.45	1.30	17.32	1.52	16.01	0.83	15.39	1.04	6.94	2.50	3.55	2.44	8.80	0.99	10.09	1.75	4.43	3.56	0.03	0.02

* ash, protein, fat, and carbohydrate contents are expressed on a dry weight basis; carbohydrates were calculated by difference; no statistically significant differences were observed between the different by-products, either between fish species or size classes.

**Table 3:** Data of mineral composition of by-product samples (head, gills, intestines, trimmings, bones, skin) of meagre and gilthead sea bream fishes in two different size classes (large and small) and the RDA and AI percentages.

By-Product Nutrient Composition																									
**Meagre**	**(mg/g)**	**Head**				**Gills**				**Intestines**				**Trimmings**				**Bones**				**Skin**			
		**Large**		**Small**		**Large**		**Small**		**Large**		**Small**		**Large**		**Small**		**Large**		**Small**		**Large**		**Small**	
		**Mean**	**SD**	**Mean**	**SD**	**Mean**	**SD**	**Mean**	**SD**	**Mean**	**SD**	**Mean**	**SD**	**Mean**	**SD**	**Mean**	**SD**	**Mean**	**SD**	**Mean**	**SD**	**Mean**	**SD**	**Mean**	**SD**
	Calcium (Ca)	5.02	0.72	8.59	1.13	5.88	0.23	6.80	0.12	0.61	0.04	0.58	0.10	12.82	0.34	46.58	1.34	6.39	0.93	9.58	0.57	5.59	0.71	3.90	0.08
	Sodium (Na)	4.07	0.37	4.92	0.56	5.52	0.13	5.92	0.27	2.53	0.12	2.12	0.57	5.87	0.99	3.72	0.43	3.46	0.11	2.76	0.73	4.42	0.28	3.15	0.17
	Potassium (K)	6.31	1.05	8.30	0.87	8.55	1.06	9.34	0.77	8.92	1.03	4.86	1.13	6.92	1.02	6.65	0.66	7.62	0.24	8.92	0.23	8.46	1.03	9.33	1.12
	Magnesium (Mg)	0.24	0.19	1.34	0.44	1.37	0.17	3.52	0.82	2.51	0.55	1.49	0.67	0.71	0.04	2.46	0.25	0.67	0.13	0.45	0.12	0.43	0.03	0.76	0.32
	% RDA/AI (Ca) *	50		86		59		68		6		6		128		466		64		96		56		39	
	% RDA/AI (Na) *	27		33		37		39		17		14		39		25		23		18		29		21	
	% RDA/AI (K) *	13		18		18		20		19		10		15		14		16		19		18		20	
	% RDA/AI (Mg) *	8		42		43		110		78		47		22		77		21		14		13		24	
**Gilthead Sea Bream**	Calcium (Ca)	8.59	0.43	5.62	1.02	4.52	0.72	7.59	0.33	0.24	0.09	0.67	0.25	42.38	0.11	11.49	0.45	9.23	0.34	7.92	1.10	2.10	0.92	0.50	0.02
	Sodium (Na)	3.68	0.21	3.28	0.21	4.26	0.82	4.54	0.72	3.01	0.79	2.39	1.01	6.81	0.35	6.08	0.26	3.43	0.14	3.79	0.99	3.52	0.13	1.34	0.22
	Potassium (K)	6.47	0.92	6.40	1.02	7.04	0.11	8.16	0.13	4.65	0.15	7.83	1.73	7.09	0.13	8.09	1.00	6.92	0.88	8.35	0.37	7.44	0.32	8.66	0.64
	Magnesium (Mg)	0.86	0.43	0.28	0.29	0.64	0.31	2.49	0.46	1.61	0.17	2.84	0.12	3.34	1.04	0.77	0.25	0.33	0.06	0.30	0.09	0.58	0.36	2.10	0.03
	% RDA/AI (Ca) *	86		56		45		76		2		7		424		115		92		79		21		5	
	% RDA/AI (Na) *	25		22		28		30		20		16		45		41		23		25		23		9	
	% RDA/AI (K) *	14		14		15		17		10		17		15		17		15		18		16		18	
	% RDA/AI (Mg) *	27		9		20		78		50		89		104		24		10		9		18		66	

* Mineral concentration was calculated as mg/g of dry matter; RDA/AI percentages for the content of 100 g of dried sample were calculated based on the following values: 1000 mg for Ca, 4700 mg for K, 329 mg for Mg, and 1500 mg for Na [31].

**Table 4:** Fatty acid content (g/100 g) of the pooled by-products from meagre and gilthead sea bream fishes.

Fatty Acids	Meagre (*Argyrosomus regius*)		Gilthead Sea Bream (*Sparus aurata*)		*p*
	Mean	SD	Mean	SD	
14:0	0.63	0.08	1.40	0.27	<0.05
15:0	0.06	0.004	0.10	0.01	<0.05
16:0	3.19	0.001	4.85	0.003	0.08
16:1	0.94	0.002	2.23	0.003	<0.001
17:0	0.05	0.01	0.08	0.02	0.19
18:0	0.71	0.01	0.78	0.05	0.91
18:1	6.63	0.001	11.34	0.01	<0.05
18:2 n-6	2.60	0.12	4.24	0.18	0.11
18:3 n-6	0.03	0.37	0.07	1.06	<0.001
18:3 n-3	0.47	0.002	0.81	0.02	<0.05
18:4 n-3	0.15	0.003	0.31	0.02	<0.05
20:0	0.07	0.07	0.09	0.21	0.39
20:1 n-9	1.14	0.03	1.53	0.09	0.32
20:2 n-9	0.02	0.01	0.09	0.02	<0.001
20:2 n-6	0.13	0.13	0.20	0.37	0.12
20:3 n-6	0.04	0.004	0.09	0.03	<0.05
20:3 n-3	0.12	0.02	0.14	0.04	0.35
20:4 n-6	0.04	0.01	0.09	0.02	<0.001
20:4 n-3	0.10	0.03	0.26	0.02	<0.001
20:5 n-3	0.23	0.01	0.42	0.02	<0.05
22:1	1.09	0.04	1.30	0.13	0.58
22:2 n-6	-	-	0.03	0.001	<0.05
23:0	0.04	0.01	0.10	0.02	<0.001
22:5 n-3	0.26	0.04	0.70	0.23	<0.001
22:6 n-3	0.30	0.05	0.53	0.15	<0.05
Total Fatty Acids	19.15	2.62	31.86	7.85	<0.05
SFA	4.84	0.69	7.45	1.71	0.05
PUFA	4.60	0.67	8.16	2.11	<0.05
MUFA	9.71	1.27	16.25	4.05	<0.05

Fatty acids are expressed as g/100 g of the lyophilized sample; *p* presents the differences between the two fish species.

**Table 5:** Protein profile data of meagre and gilthead sea bream fish by-product samples.

Meagre (*Argyrosomus regius*)			
Protein Name	Protein ID *	MW	emPAI
ATP synthase subunit epsilon. mitochondrial	XP_010742784.1	5730	4.97
NADH dehydrogenase [ubiquinone] 1 beta subcomplex subunit 1	XP_019118827.2	6895	1.75
60S ribosomal protein L35a	XP_010748069.1	12,478	1.38
cytochrome c oxidase subunit 5B. mitochondrial	XP_010737425.2	14,265	1.13
cytochrome c oxidase subunit 6B1	XP_010734575.1	10,226	1.01
U6 snRNA-associated Sm-like protein LSm2	XP_010737833.1	10,846	0.93
calcineurin subunit B type 1	XP_010728630.1	19,248	0.77
RNA-binding protein 8A isoform X1	XP_010747091.1	19,985	0.73
glutathione S-transferase omega-1	XP_010742490.3	27,659	0.7
histidine triad nucleotide-binding protein 1	XP_010741018.2	13,526	0.7
NADH dehydrogenase [ubiquinone] iron-sulfur protein 6. mitochondrial	XP_027142854.1	13,890	0.68
fatty acid-binding protein. liver	XP_010731481.1	13,998	0.67
gamma-aminobutyric acid receptor-associated protein-like 2	XP_010747638.1	14,628	0.64
prefoldin subunit 6	XP_010731190.1	14,578	0.64
acylphosphatase-2 isoform X1	XP_010745126.2	14,823	0.63
high mobility group protein B1	ADX06860.1	23,528	0.59
allograft inflammatory factor 1-like	XP_027142939.1	16,708	0.54
hypoxanthine-guanine phosphoribosyltransferase. partial	ASW22527.1	18,060	0.49
myeloid-derived growth factor	XP_019112401.2	18,073	0.49
nucleoplasmin-3	XP_027133567.1	18,296	0.49
thioredoxin domain-containing protein 12	XP_010739874.1	19,055	0.46
translationally-controlled tumor protein	XP_010747176.1	19,174	0.46
eukaryotic translation initiation factor 3 subunit G. partial	AFU54186.1	30,487	0.43
proliferation-associated protein 2G4	XP_010747198.1	43,338	0.41
glutathione peroxidase 7	XP_010740942.1	21,100	0.41
uricase	XP_010745371.2	34,496	0.38
dehydrogenase/reductase SDR family member 7C-A	XP_010739765.1	34,007	0.38
lambda-crystallin homolog isoform X1	XP_010748901.2	35,361	0.37
T-complex protein 1 subunit epsilon	XP_010731936.1	59,425	0.37
aldose reductase	XP_019116037.1	35,655	0.36
histone-binding protein RBBP4	XP_010731001.1	47,510	0.36
eukaryotic translation initiation factor 3 subunit I	XP_019115322.1	36,318	0.36
programmed cell death protein 10	XP_027130892.1	24,431	0.35
LRP chaperone MESD	XP_010746988.1	24,919	0.34
erlin-2 isoform X1	XP_027133320.1	37,660	0.34
aspartyl aminopeptidase isoform X2	XP_010748612.3	52,042	0.33
adenylate kinase 4. mitochondrial	XP_019125546.1	25,473	0.33
NADH dehydrogenase [ubiquinone] flavoprotein 2. mitochondrial	XP_019124447.1	26,706	0.32
core histone macro-H2A.1 isoform X1	XP_010735833.1	39,251	0.32
mast cell protease 1A	XP_010728896.2	26,502	0.32
RNA-binding protein FUS	XP_010735714.2	43,968	0.29
prohibitin	XP_010738397.1	29,782	0.28
aspartate—tRNA ligase. cytoplasmic	XP_019124484.1	60,564	0.28
protein kinase C and casein kinase substrate in neurons protein 3	XP_027141505.1	45,435	0.28
eukaryotic translation initiation factor 3 subunit D isoform X1	XP_027141552.1	64,192	0.26
nucleophosmin	XP_010729733.2	32,171	0.26
trimeric intracellular cation channel type A	XP_027145915.1	32,271	0.26
uncharacterized protein LOC113746832	XP_027139706.1	33,344	0.25
pollen-specific leucine-rich repeat extensin-like protein 1	XP_010728080.2	33,127	0.25
WD repeat-containing protein 61	XP_010733492.1	33,241	0.25
homogentisate 1.2-dioxygenase	XP_010754782.3	49,895	0.25
mitochondrial 2-oxodicarboxylate carrier isoform X1	XP_010742940.1	33,148	0.25
actin-related protein 2/3 complex subunit 2	XP_019118121.1	34,256	0.24
protein phosphatase 1B isoform X1	XP_010733968.1	52,276	0.24
uncharacterized protein LOC104934800	XP_010748848.3	34,731	0.24
tyrosine-protein kinase CSK	XP_010731405.1	51,060	0.24
adenylosuccinate lyase	XP_019121532.1	54,567	0.23
hydroxymethylglutaryl-CoA lyase. mitochondrial	XP_010729592.3	35,462	0.23
eukaryotic translation initiation factor 4B isoform X1	XP_010751826.3	70,327	0.23
fructose-1.6-bisphosphatase 1	XP_010746269.1	36,921	0.22
ELAV-like protein 1 isoform X1	XP_010754002.1	38,262	0.21
O-acetyl-ADP-ribose deacetylase MACROD1 isoform X1	XP_010737474.3	39,367	0.21
ankyrin repeat domain-containing protein 34C	XP_010727747.2	57,267	0.21
importin subunit alpha-3	XP_010736490.1	57,568	0.21
serine/threonine-protein phosphatase 2B catalytic subunit alpha isoform X1	XP_010752215.1	60,014	0.2
calcium-binding protein 39	XP_010729707.1	39,843	0.2
26S proteasome non-ATPase regulatory subunit 4	XP_010739959.1	40,133	0.2
alkaline phosphatase	AEL33276.1	59,799	0.2
mitochondrial cytochrome b-c1 complex subunit 8	ATN38476.1	9671	1.09
mitochondrial cytochrome c oxidase subunit 6B1 isoform A	ATN38445.1	10,139	1.02
NADH dehydrogenase [ubiquinone] 1 alpha subcomplex subunit 13	AGV76781.1	17,214	0.88
very long chain acyl-CoA synthetase. partial	AFP97557.1	46,175	0.27
mitochondrial NAD-dependent protein deacetylase sirtuin-3	AHX56275.1	48,263	0.26
carnitine palmitoyltransferase 2. partial	AUN35172.1	68,338	0.24
mitochondrial ATP synthase mitochondrial F1 complex assembly factor 1	ATN38406.1	35,887	0.23
alkaline phosphatase	AAP04486.1	57,515	0.21
cullin 5. partial	AKN80426.1	52,490	0.15
mitochondrial Rho GTPase 1-A	AGU38816.1	71,045	0.11
macrophage mannose receptor 1. partial	AIT83004.1	114,777	0.07
insulin-like growth factor-I receptor b	ALO75807.1	159,047	0.07
acetyl-CoA carboxylase alpha	ANJ04915.1	176,414	0.04

* NCBI reference sequence; emPAI score according to Ishihama et al. [34].

## Supplementary Materials

The following are available online at https://www.mdpi.com/2304-8158/9/2/190/s1, Figure S1: Histogram of head percentage per total body weight of fish, Figure S2: Histogram of gills percentage per total body weight of fish, Figure S3: Histogram of intestines percentage per total body weight of fish, Figure S4: Histogram of trimmings percentage per total body weight of fish, Figure S5: Histogram of bones percentage per total body weight of fish, Figure S6: Histogram of skin percentage per total body weight of fish.
