# Supplementary material for: Nutrient Composition and Fatty Acid and Protein Profiles of Selected Fish By-Products
Source: Foods. 2020 Feb 14;9(2):190. doi: 10.3390/foods9020190 (PMC7074476; doi:10.3390/foods9020190)
Supplement: Supplementary file 1 [file foods-09-00190-s001.zip › foods-706415-supplementary.docx]

***Supplementary material***

| 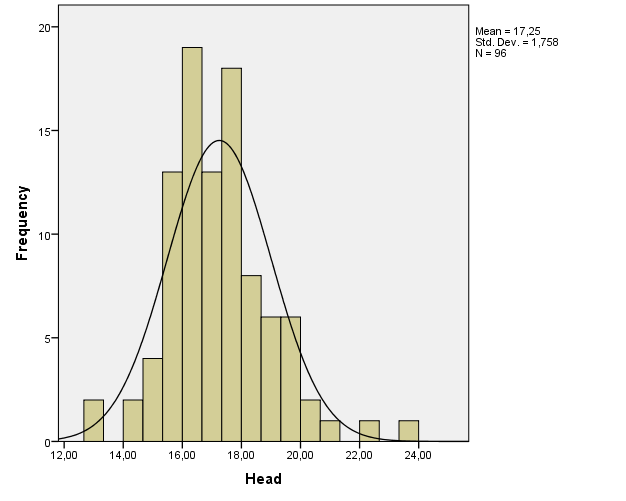 | 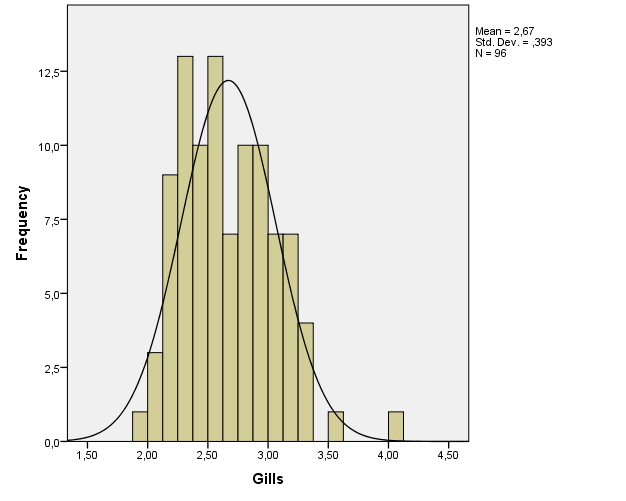 |
| --- | --- |
| Figure S1. Histogram of head percentage per total body weight of fish | Figure S2. Histogram of gills percentage per total body weight of fish |

| 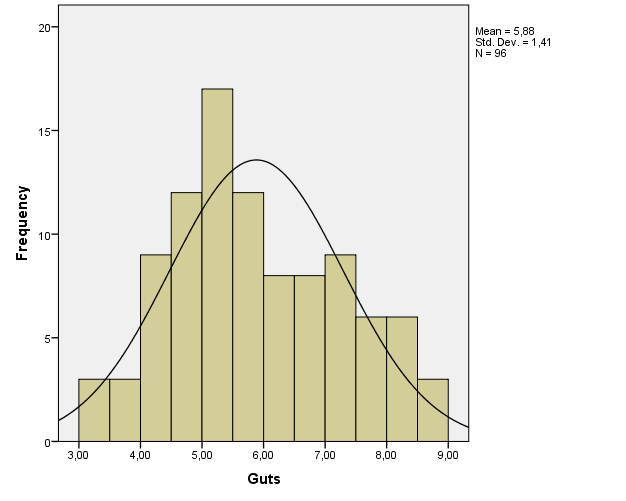 | 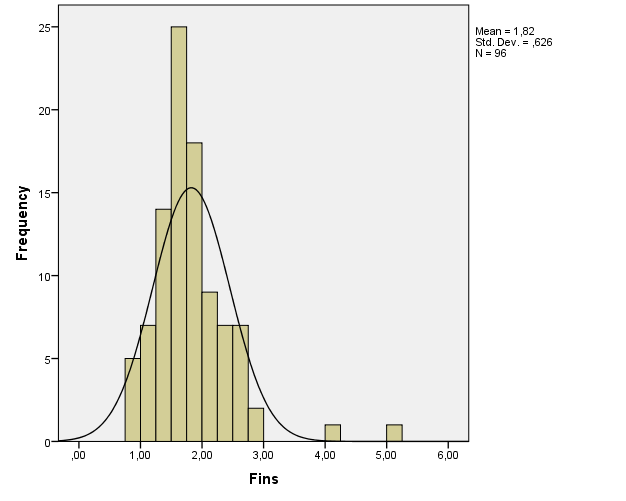 |
| --- | --- |
| Figure S3. Histogram of intestines percentage per total body weight of fish | Figure S4. Histogram of trimmings percentage per total body weight of fish |

| 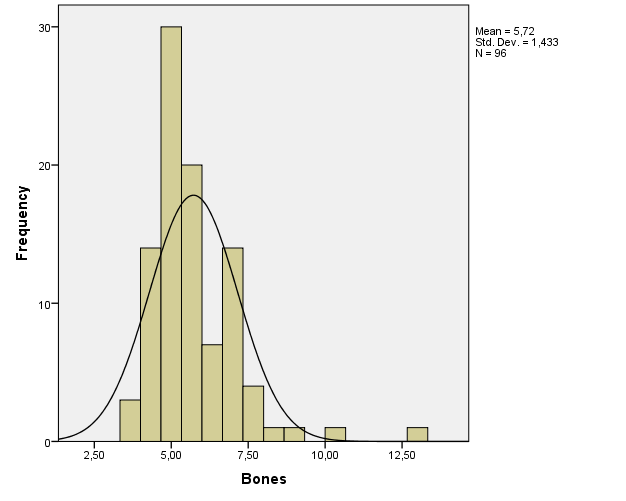 | 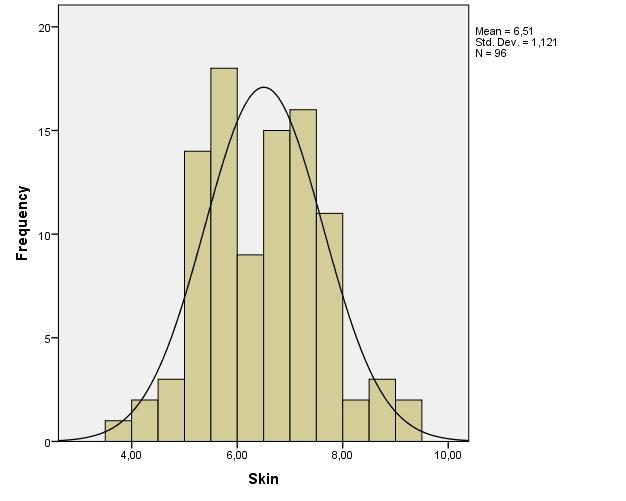 |
| --- | --- |
| Figure S5. Histogram of bones percentage per total body weight of fish | Figure S6. Histogram of skin percentage per total body weight of fish |
